# Essential growth factor receptors for fibroblast homeostasis and activation: Fibroblast Growth Factor Receptor (FGFR), Platelet Derived Growth Factor Receptor (PDGFR), and Transforming Growth Factor β Receptor (TGFβR)

**DOI:** 10.12688/f1000research.143514.2

**Published:** 2024-05-21

**Authors:** Maye F. Cheng, Faizah S. Abdullah, Matthew B. Buechler

**Affiliations:** 1 Immunology, University of Toronto, Toronto, ON, M5S 1A8, Canada

**Keywords:** Fibroblasts, growth factors, growth factor receptors, FGFR, PDGFR, TGF-b

## Abstract

Fibroblasts are cells of mesenchymal origin that are found throughout the body. While these cells have several functions, their integral roles include maintaining tissue architecture through the production of key extracellular matrix components, and participation in wound healing after injury. Fibroblasts are also key mediators in disease progression during fibrosis, cancer, and other inflammatory diseases. Under these perturbed states, fibroblasts can activate into inflammatory fibroblasts or contractile myofibroblasts. Fibroblasts require various growth factors and mitogenic molecules for survival, proliferation, and differentiation. While the activity of mitogenic growth factors on fibroblasts *in vitro* was characterized as early as the 1970s, the proliferation and differentiation effects of growth factors on these cells *in vivo* are unclear. Recent work exploring the heterogeneity of fibroblasts raises questions as to whether all fibroblast cell states exhibit the same growth factor requirements. Here, we will examine and review existing studies on the influence of fibroblast growth factor receptors (FGFRs), platelet-derived growth factor receptors (PDGFRs), and transforming growth factor β receptor (TGFβR) on fibroblast cell states.

## Introduction

Fibroblasts are non-hematopoietic cells of mesenchymal origin that are essential for the structural integrity of organs. These cells maintain tissue homeostasis and participate in diseases by secreting extracellular matrix (ECM) components and providing signalling cues for other cell types, including immune cells and other non-hematopoietic cells. ^1^ Fibroblasts are diverse and comprised of various context-specific cell states, including fibroblastic reticular cells (FRCs) in secondary lymphoid organs and peribronchial and alveolar fibroblasts in the lung. ^2^^,^^3^ The heterogeneity within fibroblasts across tissues may suggest that a progenitor-like population of fibroblasts exists within organs that can give rise to more specialized fibroblasts. ^3^ To this end, Buechler and Pradhan *et al*. demonstrated the presence of two fibroblast subsets expressing high levels of stemness-associated genes found across many tissues. ^3^ They termed these cell states as universal fibroblasts and proposed that these cells can give rise to more specialized fibroblasts, though this concept has not been unequivocally demonstrated experimentally. ^3^ It is also well-established that under certain conditions, such as injuries and cancer, fibroblasts can become activated into myofibroblasts and develop into cancer-associated fibroblasts (CAFs). ^2^^–^^4^

The phenotypic plurality of fibroblasts in both steady and diseased states highlights a challenging aspect in therapeutically targeting cells of the fibroblast lineage in fibrosis, cancer, and other inflammatory diseases. Further investigation of the signals and pathways involved in the homeostasis and activation of fibroblast subsets may open the possibility of specifically eradicating or modulating specific populations of fibroblasts. In this review, we will highlight the biology of three common growth factor receptors associated with fibroblasts and their implications for understanding fibroblasts in health and disease.

## Dermatopontin ( *Dpt ^+^*) fibroblasts may be a reservoir for specialized fibroblasts across tissues

Studying fibroblasts has traditionally been difficult given the heterogeneity of the fibroblast populations within and between tissues and a general lack of specific fibroblast markers. ^5^^,^^6^ A greater appreciation for fibroblast heterogeneity emerged with the advent of single-cell RNA-sequencing (scRNAseq). ^1^^,^^3^^,^^6^ In a differential expression analysis of data from adult murine heart, skeletal muscle, colon, and urinary bladder, a short-list of commonly expressed fibroblast markers was identified. ^6^ A total of 45 shared genes were found to be expressed within fibroblasts from these tissues, including dermatopontin ( *Dpt*) and *Peptidase inhibitor 16* ( *Pi16*). ^6^

**Figure 1.**
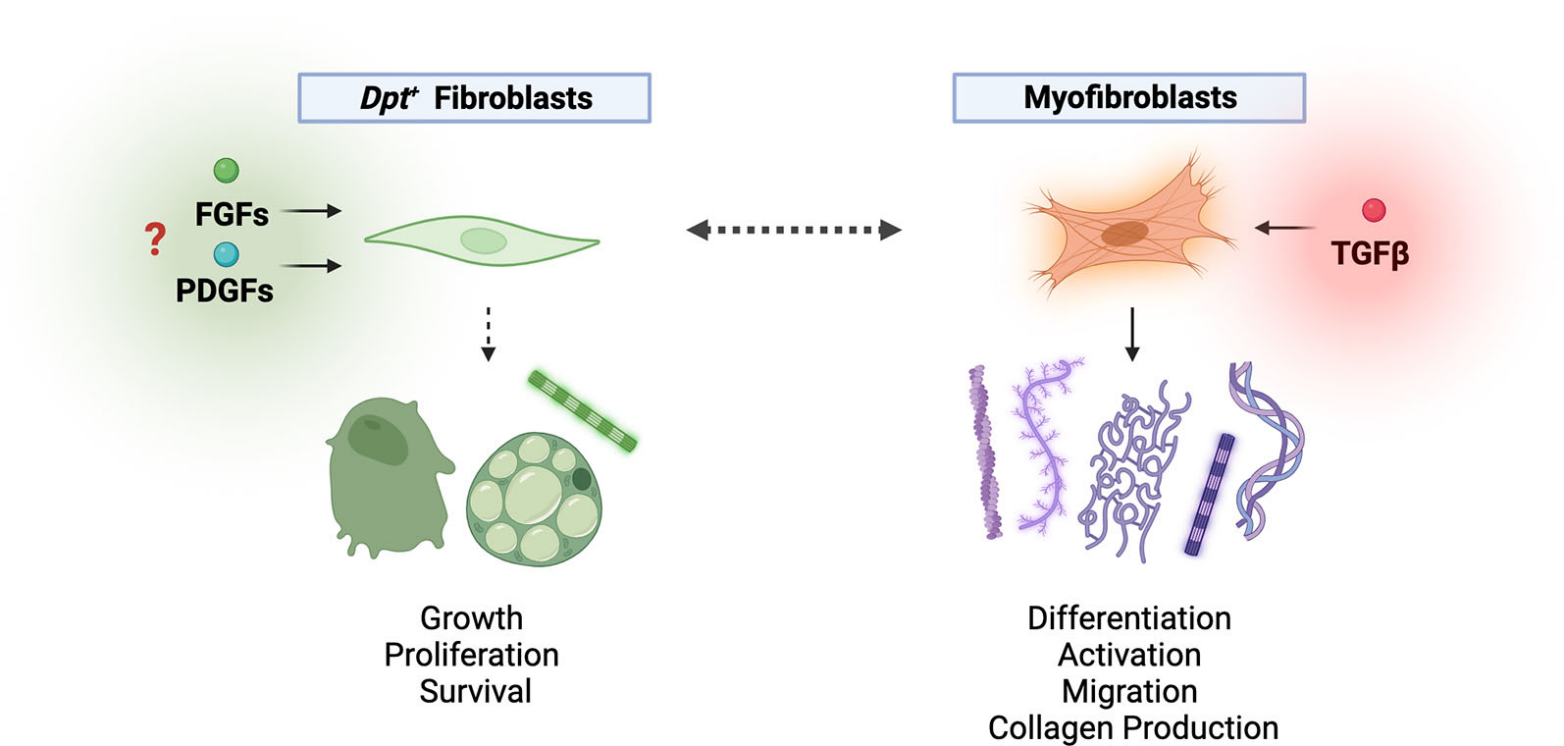
*Dpt+* fibroblasts exhibit a stem-like phenotype with signals that support homeostasis during development and steady state. Under perturbed conditions, fibroblasts can activate to become myofibroblasts with disease-specific functions. Buechler and Pradhan *et al*. identified the presence of *Dpt+* fibroblasts across multiple tissues. ^3^ These fibroblast cell states may be poised to function as early progenitors that can differentiate into various context-specific cells within the body, such as adipocytes, osteoblasts or myofibroblasts. The signals that support *Dpt+* fibroblast homeostasis have yet to be uncovered, but members of the fibroblast growth factor receptor (FGFR) and platelet-derived growth factor receptor (PDGFR) family are found to be enriched in *Dpt+* fibroblasts. ^3^ This may suggest that these signalling pathways are crucial for *Dpt+* fibroblast proliferation and survival. Myofibroblasts are more contractile cells that can produce excessive amounts of ECM components, such as α-smooth muscle actin (α-SMA) and collagen, under disease conditions. ^4^ The transition of fibroblasts to activated myofibroblasts can be dependent on the signalling cascade triggered by transforming growth factor-β (TGF-β) stimulation. Myofibroblasts are vital in wound healing and repair, although constitutive overactivation of these cells may lead to fibrosis and other disease phenotypes. ^7^^,^^8^

In a broader cross-tissue study using mouse scRNAseq data, Buechler and Pradhan *et al.* observed that two populations of fibroblasts expressing *Pi16* or *Collagen 15a1 (Col15a1)* were present in majority of the tissues examined. ^3^ These populations both displayed greater levels of stemness-associated genes and showed an enrichment for *Dpt* expression. ^3^ Due to their ubiquity across tissues, *Dpt-*enriched fibroblasts were termed ‘universal fibroblasts’ ^3^ ( Figure 1).

The presence of these fibroblasts in healthy and perturbed mouse and human tissues suggested a potential lineage trajectory involving a reservoir of *Dpt+* fibroblasts that could give rise to specialized and activated fibroblasts in the steady and diseased states, respectively. ^3^ This postulation of a universal fibroblast reservoir pool is further supported in studies examining the developmental lineage of fibroblast cell states. A study that conducted FRC-specific fate-mapping in mice identified periarterial progenitors that can give rise to splenic FRCs during embryonic development. ^9^ These embryonic progenitors express *Ly6a* and *Pdgfrα,* similar to the *Dpt+* population identified by Buechler and Pradhan *et al.* ^3^^,^^9^ The presence of *Ly6a* and *Cd34+* FRC progenitors can also be found in Peyer’s patches with the capability to replenish other FRC subsets. ^10^ Fibroblasts with this phenotype and similar progenitor-like functions were also proposed to exist in the splenic white pulp as assessed by scRNAseq. ^11^

Morphological and surface marker expression similarities between fibroblasts and mesenchymal stem/stromal cells (MSCs), a population of multipotent stem cells may prevent a clear distinction between these two cell types. ^12^^,^^13^ It has been suggested that fibroblasts represent aged MSCs or the two populations are the same, but transcriptomic analysis have proven that only distinct subsets of fibroblasts are indistinguishable from MSCs. ^14^^,^^15^ In a single-cell gene expression analysis examining the development of murine brown adipose tissue, Jun *et al. *identified a population of embryonic *Dpp4+Pi16+* fibroblasts at the onset of adipogenic development that are capable of adipogenesis. ^16^ The authors posited that this population serves as a reserve progenitor population. ^16^ Interestingly, the differentiation of *Pi16+* fibroblasts into adipocytes is akin to the adipogenic potential of MSCs. ^17^ However, further work is required to adequately define similarities and differences between MSCs and *Pi16+* fibroblasts.

Recent studies examining the fibroblast landscape in secondary lymphoid organs have also identified the presence of *Pi16+* fibroblasts among other FRCs. ^18^^,^^19^ These studies suggest that *Pi16+* fibroblasts may have immunomodulatory roles beyond functioning as a reservoir of precursor cells in the fibroblast lineage. Despite the presence of *Dpt+* fibroblasts across tissues and the mounting evidence implicating their importance in the fibroblast lineage and immunity, the signals they require for homeostasis are still poorly understood ( Figure 1). Elucidating the growth factors that sustain or expand *Dpt+* fibroblasts *in vivo* may help delineate their functional role across tissues.

## Growth factors and their signalling pathways are essential for physiological homeostasis and under pathological conditions

**Figure 2.**
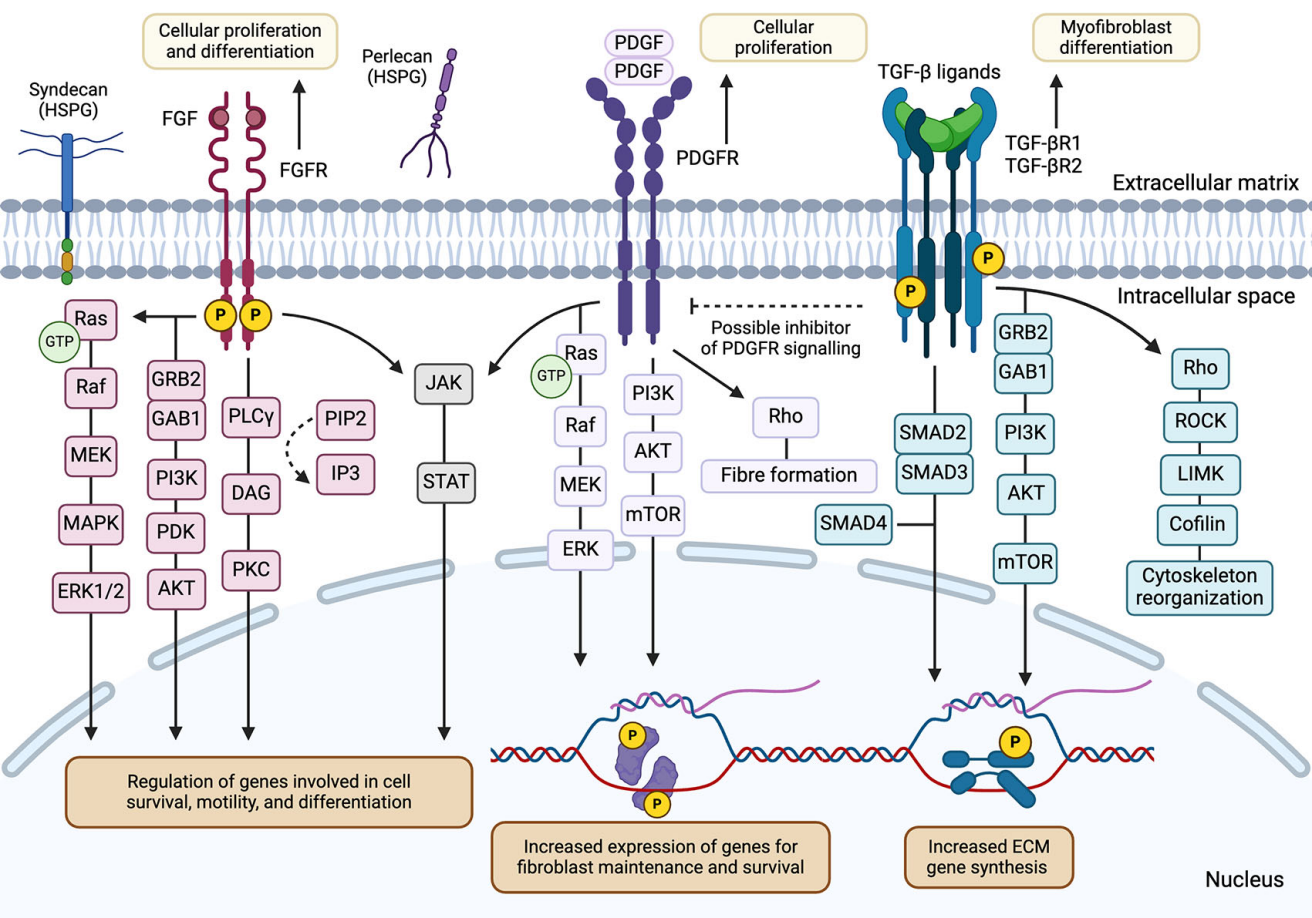
FGFR, PDGFR and TGF-β receptor signalling pathways facilitate varying context-specific downstream effects in *Dpt+* fibroblasts and myofibroblasts. Binding of respective growth factors to FGFR and PDGFR would induce receptor dimerization and activation of downstream signalling pathways, including the RAS/MAPK, PI3K-PKB/Akt, PLCγ, and JAK/STAT cascades. ^24^^–^^26^ Under physiological conditions, these pathways promote cell growth, proliferation, and survival in fibroblast populations in a context-dependent manner. TGF-β receptors may initiate the cascading phosphorylation events via SMAD proteins to induce activation, migration, and collagen production in myofibroblasts under diseased states. ^27^ Crosstalk between PDGFR and TGF-β suggests a tightly regulated system for specific biological processes, such as proliferation and differentiation, in fibroblast populations. ^28^

Growth factors and associated signalling pathways are critical for the development of mammalian tissue and cellular regeneration. The definition of growth factors broadly includes secreted molecules that regulate the cell cycle or induce cell differentiation. ^20^ Many of these growth factors also require an interaction with their corresponding cell surface receptors to trigger an intracellular signal cascade. The resulting cellular responses would include proliferation, differentiation, and gene transcription ^20^ ( Figure 2). In addition to directly impacting cellular processes, growth factors can also contribute to wound healing and tissue regeneration through a bi-directional relationship with the ECM. ^21^ The ECM can release molecules, such as heparan sulfate proteoglycans (HSPGs), which have been shown to enhance the activity of growth factors and prevent their degradation. ^22^^,^^23^ Indirectly, the ECM can also sequester cells to elicit growth factor expression and response. ^22^ Reciprocally, growth factors, such as TGF-β, may modulate ECM composition by stimulating production of ECM components or increase matrix metalloproteinases synthesis for ECM degradation. ^22^

Some of the main growth factor-dependent signalling pathways for fibroblasts are initiated by the binding of fibroblast growth factors (FGFs) and platelet-derived growth factors (PDGFs), among others including epidermal growth factors (EGFs), to their respective receptors. ^29^ These growth factors share similar downstream events involving the phosphorylation of the receptor and mediator proteins to activate combinations of intracellular signalling pathways. These conserved signalling pathways involve phosphoinositide-3-kinase protein kinase B/Akt (PI3K-PKB/Akt), the mitogen-activated protein kinase (MAPK), phospholipase C γ (PLC γ) cascades, and transcription factors that include the signal transducers and activators of transcription (STATs) or SMAD proteins ^24^^–^^26^^,^^29^ ( Figure 2). The subsequent biological effects of these growth factors are influenced by several parameters, including the concentration of the growth factor and the presence of other stimuli. ^30^ The different circumstances impacting growth factor signalling functions may depend on the specific tissue and cell type implicated. For example, a member of the FGF family, FGF2, can promote cell proliferation in cell types such as pancreatic stellate cells, neural crest cells and MSCs. ^31^^–^^34^ However, FGF2 is also found to play a role in endothelial cell migration and may promote osteocyte differentiation in MSCs. ^35^^–^^37^ The tissue and cell-specific activity of growth factors allow for more targeted biological functions under normal, physiological conditions.

Growth factors are crucial for maintaining homeostasis under healthy conditions; however, they have also been implicated to elicit disease progression. For example, during the transformative process from normal cells to malignant cancer cells, numerous genetic mutations accumulate. ^38^ Usually, these mutations involve the loss of tumour suppressor gene functions or incite oncogene functions, which would eventually lead to failure of DNA repair mechanisms. ^38^ Once premalignant cells begin to accumulate these oncogenic mutations, they can proliferate and clonally expand by the activation of signalling pathways orchestrated by growth factors. ^38^ The expanded cancer cells would eventually migrate and penetrate adjacent tissues, contributing to metastases. During this epithelial-to-mesenchymal transition (EMT), malignant cells would engage the transcription of a mesenchymal genetic program, promoting a transition from exhibiting epithelial features to acquiring mobility as mesenchymal cells. ^39^ This progressive conversion is also supported and mediated by growth factors, such as TGF-β, FGF, and EGF. ^38^ In addition to signalling cancer cells directly, growth factors, such as TGF-β, can also influence the surrounding cancer microenvironment by enriching the presence of myofibroblasts and CAFs. ^40^ In later stages of disease, cancer cells depend on angiogenesis for metastasis and tumour growth, which is further stimulated by mitogenic growth factors including FGF and vascular endothelial growth factor (VEGF). ^38^

Growth factor-based therapeutic strategies for cancer, fibrosis, and other diseases may have systemic implications due to their wide range of effects in the body. The majority of transgenic animal models with genes eliminated from growth factor family members are embryonically or postnatally lethal. ^38^ Targeting components of aberrant pathways that are causing uncontrolled proliferation or differentiation rather than the growth factors themselves may be a more feasible therapeutic option. Therefore, abrogating the interacting growth factor receptors may provide more focused therapeutic targets. As the first point of contact in the signalling pathway and a key transducer of the mitogenic signal, disrupting receptor activation may yield a more specific response than targeting the downstream signalling cascades shared between multiple growth factors and pathways. Further investigating the biological implications of FGFRs, PDGFRs, and TGFβR on fibroblast homeostasis may augment the efficacy of current treatments for fibrotic diseases.

## Fibroblast growth factor receptors

Fibroblast growth factors (FGFs) were first identified in the 1970s following the discovery that a macromolecule isolated from the pituitary and the brain enhanced the growth of 3T3 cells, a mouse fibroblast cell line. ^41^ Since its discovery, this macromolecule named a “fibroblast growth factor” has demonstrated its importance in mediating fundamental processes during embryonic development through to adulthood. ^24^^,^^42^ Currently, 23 different FGFs have been identified, with the majority signalling through one of four fibroblast growth factor receptors (FGFRs) – FGFR1, FGFR2, FGFR3, and FGFR4. ^24^ These highly conserved isoforms of FGFR vary based on alternative splicing of the transcripts. ^42^ The differences between each FGFR extracellular domain profoundly affect the specific ligand-binding ability of each receptor. ^42^ Additionally, it has been shown that the expression of FGFR isoforms is dependent on cell type and tissue. ^42^ For example, the FGFR2b isoform is only expressed in epithelial cells, while FGFR2c is exclusively expressed in mesenchymal cells. ^42^ Despite differences in FGFR isoform expression across cell types, FGFR signalling can be detected in all human tissues at varying levels. ^43^

Similar to other members of receptor tyrosine kinase (RTK) families, FGFRs are single-pass transmembrane proteins that dimerize upon FGF binding. ^24^^,^^25^ Canonical FGF signal transduction through FGFR is assisted by heparin and heparan sulfate proteoglycan cofactors. ^23^ Alternatively, activation of FGFR by endocrine FGFs require Klotho co-receptors as cofactors. ^24^^,^^44^ After dimerization and autophosphorylation of tyrosine residues in the cytoplasmic region of the receptor, various downstream signalling pathways activate ( Figure 2). Thus, initiating physiological functions including cellular proliferation and differentiation, angiogenesis, and wound healing. ^26^

## Fibroblast growth factor receptors can induce proliferation and differentiation in fibroblast populations

As potent mitogenic receptors, the FGFR signal transduction pathway is tightly regulated by a negative feedback loop under normal physiological conditions. ^45^ Under circumstances of aberrant FGFR activation, the resulting uncontrolled mitogenic effects contribute to 5-10% of all human cancers. ^46^ The ability of FGFR to induce proliferation and differentiation is suggested in many cell types, especially in fibroblasts, where it was first discovered. An example of FGFR’s role in proliferation is demonstrated from FGFR1 inhibition in MSCs. ^47^ The loss of FGFR1 signalling results in a decrease in MSC expansion, a complete halt in the cell cycle, and has a negative impact on early mesoderm development. ^47^ Taken together, these data suggest FGFR1 signalling may play a role in regulating stemness during proliferation and lineage-commitment in MSCs. ^47^ In models of adipogenesis, inhibition of FGFR1 leads to a decrease in both FGF-1-mediated proliferation and priming for differentiation in human adipose fibroblasts. ^48^ Similarly, Xu and Dai demonstrated that mice with a fibroblast-specific ablation of FGFR2 displayed a decrease in interstitial cell proliferation and apoptosis after being challenged with ischemia/reperfusion injury to induce kidney damage. ^49^ The improvement in kidney fibrosis in the FGFR2 knock-out mice suggests that this was observed due to the inhibition of kidney fibroblast proliferation and activation. ^49^

The mitogenic effects of FGFR signalling have been examined extensively, however, its pro-proliferative phenotype may be tissue and cell-specific. In a paper examining FGFR1 signalling in basal cells of adult mouse trachea, Balasooriya *et al.* reported that loss of FGFR1 signalling increased levels of lung basal cell proliferation. ^50^ This observation contrasts the previously reported mitogenic effects of FGFR signalling in fibroblasts. It is suggested that lack of FGFR1 signalling prevents post-translational activation of sprouty RTK signaling antagonist 2 (SPRY2), a protein known to inhibit intracellular signalling downstream of other RTKs in basal cells. ^50^ This finding affirms the complexity of examining individual growth factor receptors, where context-dependent and compensatory effects from other signalling pathways may prevent a clear phenotype from being observed. Future research on FGFR signalling should prioritize the development of mouse models that can selectively delete growth factor receptor expression on fibroblasts. This will better recapitulate and distill the effects of growth factor receptors and delineate tissue or cell-specific effects in an *in vivo* environment.

The role of FGFR signalling in *Dpt+* universal fibroblasts has yet to be addressed. However, the implications of FGFR signalling in early embryogenesis and their effects on MSC differentiation point to its importance for the homeostasis of fibroblast progenitor cells. ^47^^,^^51^^,^^52^ It is tempting to speculate that autocrine or paracrine FGF2 may enable self-renewal or homeostasis of tissue-wide progenitor fibroblast population *in vivo*, much like pathways that have been proposed to underlie MSC homeostasis. ^47^^,^^51^^,^^52^ Interestingly, *Fgfr1* expression was uniquely enhanced in universal fibroblast clusters, but not other members of the FGFR family. ^3^ Therefore, a greater understanding of *Dpt*+ fibroblast subsets may reveal differential *Fgfr* expression and further elucidate the signals required by these fibroblast progenitor cells *in vivo.*

## Platelet-derived growth factor receptors

Platelet-derived growth factors, or PDGFs, have been shown to play an integral role in fibroblast biology. ^53^^–^^56^ It is now appreciated that there are five isoforms of PDGF: the four homodimers PDGF-AA, PDGF-BB, PDGF-CC, PDGF-DD, and the heterodimer PDGF-AB. ^57^ These ligands function by binding to two receptors, referred to as platelet-derived growth factor receptor-α (PDGFRα) and platelet-derived growth factor receptor-β (PDGFRβ), which are broadly expressed among mesenchymal cell types, including fibroblasts. ^57^ These receptors dimerize following ligand binding, allowing for autophosphorylation, signal transduction, and the invocation of ubiquitous signalling cascades, such as the JAK/STAT and PI3K/Akt pathways ^58^^,^^59^ ( Figure 2). In turn, PI3K/Akt signalling can recruit mediators that are involved in cellular proliferation and survival, such as intracellular components of the Erk/MAPK pathway ^57^^–^^59^ ( Figure 2). Initial purification of PDGFRα through the cloning of murine cDNA encoding the receptor identified conserved features, such as a transmembrane domain, extracellular cystine residues, and a tyrosine kinase domain. ^60^ The receptor may undergo a variety of post-translational modifications prior to its expression on the membrane, including O-linked oligosaccharide and ubiquitin addition. ^60^ After ligand binding and downstream signal transduction and amplification, both PDGFRα and PDGFRβ can moderate proliferation, differentiation, survival, and chemotaxis, among other key cellular effects. ^53^^,^^57^^–^^59^

## Signalling through platelet-derived growth factor receptors promote fibroblast maintenance and proliferation

In the mid-1970s, platelets from serum were found to enhance growth in 3T3 fibroblasts *in vitro.* ^61^ These factors, later identified as PDGFs, allow for cellular stimulation and subsequent proliferation upon binding to their receptors. ^61^ The implication of PDGFR signalling on fibroblast proliferation is demonstrated by transgenic mouse models with conditional knock-ins of PDGFRα. Primary embryonic fibroblasts isolated from murine embryos with constitutive PDGFRα signalling activity display greater proliferative ability compared to wild type embryos. ^53^ Additionally, adult mice with constitutive PDGFRα signalling display aberrant levels of fibroblast activity, including connective tissue hyperplasia and excessive ECM production. ^53^ The resulting fibrosis and tissue scarring phenotype signifies the importance of PDGFRα in supporting key fibroblast functions. ^53^

Animal models with deficient PDGFRα expression further support its importance for fibroblast maintenance and survival. In an inactive state, cardiac fibroblasts are often quiescent and undergo limited proliferation. ^54^ A tamoxifen-induced deletion of PDGFRα in cells expressing the cardiac fibroblast-specific transcription factor 21 ( *Tcf21 ^mCrem^*) results in a drastic loss in ventricular cardiac fibroblasts *in vivo.* ^54^ This implies the need for a basal level of PDGFRα signalling to maintain resident cardiac fibroblast populations. ^54^ It is hypothesized that basal levels of PDGFRα signalling may prevent apoptotic signals that would lead to cell death as represented by a reduction in cell numbers. ^54^ Asli *et al.* also reported that PDGFRα inhibition in a stem cell-like population of cardiac fibroblasts *in vitro* demonstrates limited rates of self-renewal. ^55^ However, an enhanced synthesis of ribosomal and ribosomal-related genes, such as *Eif1*, *Eif2s1*, and *Eif4a1,* is observed following *in vivo* PDGF-AB treatment. ^55^ Taken together, these data suggest that signalling via the PDGFRα pathway may prompt fibroblasts to exit quiescence and instead enter a translationally active state.

In addition to promoting fibroblast biological processes in steady state, PDGFR signalling may play a role during wound healing. An inhibition of wound closure is observed in scratch-wound assays where dermal fibroblasts lack PDGFRβ expression. ^56^ This may suggest that PDGFRβ signalling is vital to fibroblast migration and proliferation. ^56^ *Ex vivo* stimulation of adipocyte precursor (AP) cells with PDGF-CC results in an increase in their proliferation, while *in vivo* injections of PDGF-CC-neutralizing antibodies decrease AP cell numbers. ^62^ While PDGF-CC is indispensable for the expansion of the AP cell population, it is not required for the development of other myofibroblast subsets during wound healing. ^62^ Shook *et al.* demonstrated that local injections of PDGF-CC-neutralizing antibodies in wounds does not result in significant changes in the proliferation of non-AP myofibroblast subsets, nor in general wound re-revascularization during healing. ^62^

Notably, the expansion of precursor fibroblast populations after PDGFR activation via PDGF-AB and PDGF-CC binding may imply the importance of PDGFR signalling in the *Dpt+* fibroblast population. ^55^^,^^62^ To this end, transgenic animal models with selective ablation of PDGFR signalling in *Dpt+* fibroblasts may elucidate the importance of this receptor on the proliferation, survival, and activation of universal fibroblasts. Altogether, downstream pathways stimulated through PDGFRα and β signalling may be essential for fibroblast precursor cell states to exit a state of quiescence and activate biological processes, such as cell proliferation, self-renewal, migration, and apoptosis.

## Fibroblasts depend on TGF-β receptor signalling for differentiation into myofibroblasts

While fibroblasts participate in essential functions including ECM remodelling and structural support during homeostasis, they can also become activated and transform into myofibroblasts under diseased conditions. In this state, cells can produce several compounds, such as α-SMA, ECM fibers, and collagen. ^7^ The excessive production of these molecules can then further promote the activation of myofibroblasts in a positive feedback loop, resulting in an uncontrolled, pathological fibrotic state. ^63^ There is a drastic increase in ECM production following the establishment of myofibroblasts in the damaged tissue. ^63^ Following this, signals within the wound bed can either trigger the cells to transform into a fibrotic phenotype or enter a quiescent state and eventually commit apoptosis. ^64^ Loss of physical stresses would prompt the cells to undergo cell death, while persistent mechanical tension would promote hypertrophic scar formation through the inhibition of apoptosis. ^64^^,^^65^ Persistent collagen secretion and fibrotic activity can lead to excessive scar formation and conditions with high disease burdens, such as idiopathic pulmonary fibrosis. ^66^

There are several factors in the microenvironment that can stimulate the transition of conventional fibroblasts to activated myofibroblasts, including physical and biochemical stresses and ECM remodelling. ^64^ Microenvironmental changes during inflammation also lead to the recruitment and infiltration of immune cells and the subsequent release of cytokines, such as IL-4, IL-13, tumor necrosis factor-alpha (TNF-α), and TGF-β. ^27^^,^^67^^–^^69^ Members of the tumor necrosis super family (TNFSF), which include TNF-α (TNFSF2), are also known proinflammatory mediators with implications in promoting fibroblast activation and have been reviewed elsewhere. ^70^^,^^71^

It has been well-established that TGF-β is a crucial mediator in the transition from fibroblasts to myofibroblasts. TGF-β, which exists in three isoforms (TGF-β1, TGF-β2, and TGF-β3), activates homo- or heterodimeric complexes consisting of the type I TGF-β receptor (TβRI) and the type II TGF-β receptor (TβRII) ^27^ ( Figure 2). Subsequent transcriptional changes can be exerted through a cascade of phosphorylation events involving the SMAD protein family ^27^ ( Figure 2). Fibroblasts cultured in the presence of TGF-β have demonstrated increased levels of myofibroblast-associated molecules, such as α-SMA, procollagen I-α-1, and ED-A fibronectin. ^72^^,^^73^ Alternatively, human fibroblasts treated with TGF-β and Lovastatin, a TGF-β inhibitor, prevented the transition of fibroblasts to myofibroblasts. ^74^

The production of TGF-β and associated cytokines from proximal immune cells is a key contributor to the fibroblast to myofibroblast transition. The release of TGF-β and IL-4 from M2 macrophages in the surrounding environment induces a myofibroblast-specific transcriptional state through the phosphorylation of SMAD3 and the induction of the JAK/STAT and PI3K/Akt signalling cascades ^67^ ( Figure 2). Additionally, co-culture of macrophages with human vocal fold fibroblasts has been associated with increased levels of pro-fibrotic compounds, including type I collagen and α-SMA. ^75^ This observation complements the noted amplification of genes involved in ECM productions, including *Acta2* and *Col1a1*. ^75^ The co-culture of fibroblasts with eosinophils also increased the expression of α-SMA through the stimulation of latent TGF-β and upregulated the transcription of fibronectin and collagen; thus, inducing the fibroblast to myofibroblast transition. ^76^^,^^77^

In addition to TGF-β mediating the transition of fibroblasts to myofibroblasts, PDGFR signalling may also activate fibroblasts in a context-dependent manner. The overexpression of PDGFR ligands, PDGF-AA and PDGF-BB, has demonstrated varying severity of cardiac fibrotic phenotypes in murine models. ^78^ This difference in fibrotic phenotypes may be due to the variation in binding affinity between the ligands and PDGFRα. ^78^ Furthermore, the loss of PDGFRα and PDGFRβ in transgenic murine models results in a reduction in the number of differentiated epicardial-derived cardiac fibroblasts, suggesting that PDGFR signalling is essential for the differentiation and activation of fibroblast subsets. ^78^^,^^79^

These data indicate that TGF-β may behave in a morphogenic fashion, with its levels balancing myofibroblast and tissue-specific fibroblast levels within the body. ^28^ TGF-β may act in a negative feedback loop with PDGFRα to prevent negative fibrotic outcomes. Increased levels of TGF-β favour myofibroblast differentiation, thus a corresponding decrease in PDGFR expression may limit potential fibrosis driven by fibroblast proliferation. As such, it would be valuable to explore the effects of TGF-β on *Dpt+* fibroblasts and elucidate the potential crosstalk between TGF-β and PDGFR or other growth factor receptors on this fibroblast cell state.

## Conclusion

Fibroblasts are found throughout organs across species, yet knowledge of these cells remain elusive. Recent scRNA-seq approaches have suggested that a hierarchy of transcriptional cell states exist within the fibroblast lineage. The role of growth factor receptors in proliferation and maintenance of various cell types is well-characterized, but their functions in discrete fibroblasts cell states remain unclear. Transgenic animal models with modified or eliminated growth factor receptor expressions in fibroblast subsets would be valuable for deciphering the signals required by fibroblasts under different conditions. However, the downstream signalling cascades of receptor tyrosine kinases are commonly shared by multiple growth factor receptors. This can pose challenges in isolating effects from individual growth factor receptors when examining signalling requirements for fibroblasts in *in vitro* and *in vivo* models. Furthermore, current literature provides an extensive catalog of growth factor receptors and activation signals that may have implications in fibroblast maintenance and differentiation. In this review, three common growth factor receptors associated with fibroblast homeostasis were explored, but this does not dismiss the contribution of other signalling pathways, which should be addressed in future studies. Nonetheless, exploring growth factor receptor signalling activity within the cell states that comprise the fibroblast lineage will promote the development of more specific and targeted therapies for cancer, fibrosis, and other inflammatory diseases.

## Data Availability

No data are associated with this article.

## References

[1] Plikus MV : Fibroblasts: Origins, definitions, and functions in health and disease. *Cell.* 2021;184:3852–3872. 10.1016/j.cell.2021.06.024 34297930 PMC8566693

[2] Sherman MH Magliano MP di : Cancer-Associated Fibroblasts: Lessons from Pancreatic Cancer. *Annu. Rev. Cancer Biol.* 2023;7:43–55. 10.1146/annurev-cancerbio-061421-035400

[3] Buechler MB : Cross-tissue organization of the fibroblast lineage. *Nature.* 2021;593:575–579. 10.1038/s41586-021-03549-5 33981032

[4] Otranto M : The role of the myofibroblast in tumor stroma remodeling. *Cell Adhes. Migr.* 2012;6:203–219. 10.4161/cam.20377 22568985 PMC3427235

[5] Janmaat CJ : Human Dermal Fibroblasts Demonstrate Positive Immunostaining for Neuron- and Glia- Specific Proteins. *PLoS One.* 2015;10:e0145235. 10.1371/journal.pone.0145235 26678612 PMC4683011

[6] Muhl L : Single-cell analysis uncovers fibroblast heterogeneity and criteria for fibroblast and mural cell identification and discrimination. *Nat. Commun.* 2020;11:3953. 10.1038/s41467-020-17740-1 32769974 PMC7414220

[7] D’Urso M Kurniawan NA : Mechanical and Physical Regulation of Fibroblast–Myofibroblast Transition: From Cellular Mechanoresponse to Tissue Pathology. *Front. Bioeng. Biotechnol.* 2020;8:609653. 10.3389/fbioe.2020.609653 33425874 PMC7793682

[8] Younesi FS Miller AE Barker TH : Fibroblast and myofibroblast activation in normal tissue repair and fibrosis. *Nat. Rev. Mol. Cell Biol.* 2024;1–22. 10.1038/s41580-024-00716-0 38589640

[9] Cheng H-W : Origin and differentiation trajectories of fibroblastic reticular cells in the splenic white pulp. *Nat. Commun.* 2019;10:1739. 10.1038/s41467-019-09728-3 30988302 PMC6465367

[10] Prados A : Fibroblastic reticular cell lineage convergence in Peyer’s patches governs intestinal immunity. *Nat. Immunol.* 2021;22:510–519. 10.1038/s41590-021-00894-5 33707780 PMC7610542

[11] Alexandre YO : A diverse fibroblastic stromal cell landscape in the spleen directs tissue homeostasis and immunity. *Sci. Immunol.* 2022;7:eabj0641. 10.1126/sciimmunol.abj0641 34995096

[12] Bae S : Gene and microRNA expression signatures of human mesenchymal stromal cells in comparison to fibroblasts. *Cell Tissue Res.* 2008;335:565. 19089456 10.1007/s00441-008-0729-y

[13] Denu RA : Fibroblasts and Mesenchymal Stromal/Stem Cells Are Phenotypically Indistinguishable. *Acta Haematol.* 2016;136:85–97. 10.1159/000445096 27188909 PMC4988914

[14] Budeus B Unger K Hess J : Comparative computational analysis to distinguish mesenchymal stem cells from fibroblasts. *Front. Immunol.* 2023;14:1270493. 10.3389/fimmu.2023.1270493 37822926 PMC10562561

[15] Fan C : Single-Cell Transcriptome Integration Analysis Reveals the Correlation Between Mesenchymal Stromal Cells and Fibroblasts. *Front. Genet.* 2022;13: 798331. 10.3389/fgene.2022.798331 35360851 PMC8961367

[16] Jun S : Control of murine brown adipocyte development by GATA6. *Dev. Cell.* 2023;58:2195–2205.e5. 10.1016/j.devcel.2023.08.003 37647897 PMC10842351

[17] Kim J Ko J : A novel PPARγ2 modulator sLZIP controls the balance between adipogenesis and osteogenesis during mesenchymal stem cell differentiation. *Cell Death Differ.* 2014;21:1642–1655. 10.1038/cdd.2014.80 24948012 PMC4158692

[18] Martin AD : PI16+ reticular cells in human palatine tonsils govern T cell activity in distinct subepithelial niches. *Nat. Immunol.* 2023;24:1138–1148. 10.1038/s41590-023-01502-4 37202490 PMC10307632

[19] Lütge M : Conserved stromal–immune cell circuits secure B cell homeostasis and function. *Nat. Immunol.* 2023;24:1149–1160. 10.1038/s41590-023-01503-3 37202489 PMC10307622

[20] Stone WL Leavitt L Varacallo M : Physiology, Growth Factor. *StatPearls.* 2023. 28723053

[21] Kulebyakin KY Nimiritsky PP Makarevich PI : Growth Factors in Regeneration and Regenerative Medicine: “the Cure and the Cause.”. *Front. Endocrinol.* 2020;11:384. 10.3389/fendo.2020.00384 32733378 PMC7358447

[22] Schultz GS Wysocki A : Interactions between extracellular matrix and growth factors in wound healing. *Wound Repair Regen.* 2009;17:153–162. 10.1111/j.1524-475X.2009.00466.x 19320882

[23] Sarrazin S Lamanna WC Esko JD : Heparan Sulfate Proteoglycans. *Cold Spring Harb. Perspect. Biol.* 2011;3:a004952. 21690215 10.1101/cshperspect.a004952 PMC3119907

[24] Hui Q Jin Z Li X : FGF Family: From Drug Development to Clinical Application. *Int. J. Mol. Sci.* 2018;19:1875. 10.3390/ijms19071875 29949887 PMC6073187

[25] Sarabipour S Hristova K : Mechanism of FGF receptor dimerization and activation. *Nat. Commun.* 2016;7:10262. 10.1038/ncomms10262 26725515 PMC4725768

[26] Furdui CM Lew ED Schlessinger J : Autophosphorylation of FGFR1 Kinase Is Mediated by a Sequential and Precisely Ordered Reaction. *Mol. Cell.* 2006;21:711–717. 10.1016/j.molcel.2006.01.022 16507368

[27] Attisano L Wrana JL : Signal Transduction by the TGF-β Superfamily. *Science.* 2002;296:1646–1647. 10.1126/science.1071809 12040180

[28] Contreras O : Cross-talk between TGF-β and PDGFRα signaling pathways regulates the fate of stromal fibro–adipogenic progenitors. *J. Cell Sci.* 2019;132:jcs232157. 31434718 10.1242/jcs.232157

[29] Rodrigues M Griffith LG Wells A : Growth factor regulation of proliferation and survival of multipotential stromal cells. *Stem Cell Res. Ther.* 2010;1:32. 10.1186/scrt32 20977782 PMC2983445

[30] Cross M Dexter TM : Growth factors in development, transformation, and tumorigenesis. *Cell.* 1991;64:271–280. 10.1016/0092-8674(91)90638-F 1988148

[31] Coleman SJ : Nuclear translocation of FGFR1 and FGF2 in pancreatic stellate cells facilitates pancreatic cancer cell invasion. *EMBO Mol. Med.* 2014;6:467–481. 10.1002/emmm.201302698 24503018 PMC3992074

[32] Murphy M Reid K Ford M : FGF2 regulates proliferation of neural crest cells, with subsequent neuronal differentiation regulated by LIF or related factors. *Development.* 1994;120:3519–3528. 10.1242/dev.120.12.3519 7821219

[33] Teixeira BL Amarante-Silva D Visoni SB : FGF2 Stimulates the Growth and Improves the Melanocytic Commitment of Trunk Neural Crest Cells. *Cell. Mol. Neurobiol.* 2020;40:383–393. 10.1007/s10571-019-00738-9 31555941 PMC11448768

[34] Ahn H-J Lee W-J Kwack K : FGF2 stimulates the proliferation of human mesenchymal stem cells through the transient activation of JNK signaling. *FEBS Lett.* 2009;583:2922–2926. 10.1016/j.febslet.2009.07.056 19664626

[35] Byun MR : FGF2 stimulates osteogenic differentiation through ERK induced TAZ expression. *Bone.* 2014;58:72–80. 10.1016/j.bone.2013.09.024 24125755

[36] Michaelis UR : Mechanisms of endothelial cell migration. *Cell. Mol. Life Sci.* 2014;71:4131–4148. 10.1007/s00018-014-1678-0 25038776 PMC11113960

[37] Xiao L : Disruption of the Fgf2 gene activates the adipogenic and suppresses the osteogenic program in mesenchymal marrow stromal stem cells. *Bone.* 2010;47:360–370. 10.1016/j.bone.2010.05.021 20510392 PMC2947437

[38] Witsch E Sela M Yarden Y : Roles for Growth Factors in Cancer Progression. *Physiology.* 2010;25:85–101. 10.1152/physiol.00045.2009 20430953 PMC3062054

[39] Roche J : The Epithelial-to-Mesenchymal Transition in Cancer. *Cancers.* 2018;10:52. 10.3390/cancers10020052 29462906 PMC5836084

[40] Kalluri R : The biology and function of fibroblasts in cancer. *Nat. Rev. Cancer.* 2016;16:582–598. 10.1038/nrc.2016.73 27550820

[41] Gospodarowicz D : Localisation of a fibroblast growth factor and its effect alone and with hydrocortisone on 3T3 cell growth. *Nature.* 1974;249:123–127. 10.1038/249123a0 4364816

[42] Eswarakumar VP Lax I Schlessinger J : Cellular signaling by fibroblast growth factor receptors. *Cytokine Growth Factor Rev.* 2005;16:139–149. 10.1016/j.cytogfr.2005.01.001 15863030

[43] Hughes SE : Differential Expression of the Fibroblast Growth Factor Receptor (FGFR) Multigene Family in Normal Human Adult Tissues. *J. Histochem. Cytochem.* 1996;45:1005–1019. 10.1177/002215549704500710 9212826

[44] Liu G : Inhibition of FGF-FGFR and VEGF-VEGFR signalling in cancer treatment. *Cell Prolif.* 2021;54:e13009. 10.1111/cpr.13009 33655556 PMC8016646

[45] Szybowska P Kostas M Wesche J : Negative Regulation of FGFR (Fibroblast Growth Factor Receptor) Signaling. *Cells.* 2021;10:1342. 10.3390/cells10061342 34071546 PMC8226934

[46] Xian W Schwertfeger KL Vargo-Gogola T : Pleiotropic effects of FGFR1 on cell proliferation, survival, and migration in a 3D mammary epithelial cell model. *J. Cell Biol.* 2005;171:663–673. 10.1083/jcb.200505098 16301332 PMC2171554

[47] Dombrowski C : FGFR1 Signaling Stimulates Proliferation of Human Mesenchymal Stem Cells by Inhibiting the Cyclin-Dependent Kinase Inhibitors p21Waf1 and p27Kip1. *Stem Cells.* 2013;31:2724–2736. 10.1002/stem.1514 23939995 PMC8553008

[48] Widberg CH : Fibroblast growth factor receptor 1 is a key regulator of early adipogenic events in human preadipocytes. *Am. J. Physiol.-Endocrinol. Metab.* 2009;296:E121–E131. 10.1152/ajpendo.90602.2008 18940940

[49] Xu Z Dai C : Ablation of FGFR2 in Fibroblasts Ameliorates Kidney Fibrosis after Ischemia/Reperfusion Injury in Mice. *Kidney Dis.* 2017;3:160–170. 10.1159/000484604 29344510 PMC5757553

[50] Balasooriya GI Johnson J-A Basson MA : An FGFR1-SPRY2 Signaling Axis Limits Basal Cell Proliferation in the Steady-State Airway Epithelium. *Dev. Cell* 2016;37:85–97. 10.1016/j.devcel.2016.03.001 27046834 PMC4825408

[51] Dorey K Amaya E : FGF signalling: diverse roles during early vertebrate embryogenesis. *Development.* 2010;137:3731–3742. 10.1242/dev.037689 20978071 PMC3747497

[52] Kähkönen TE : Role of fibroblast growth factor receptors (FGFR) and FGFR like-1 (FGFRL1) in mesenchymal stromal cell differentiation to osteoblasts and adipocytes. *Mol. Cell. Endocrinol.* 2018;461:194–204. 10.1016/j.mce.2017.09.015 28923346

[53] Olson LE Soriano P : Increased PDGFRα Activation Disrupts Connective Tissue Development and Drives Systemic Fibrosis. *Dev. Cell.* 2009;16:303–313. 10.1016/j.devcel.2008.12.003 19217431 PMC2664622

[54] Ivey MJ Kuwabara JT Riggsbee KL : Platelet-derived growth factor receptor-α is essential for cardiac fibroblast survival. *Am. J. Physiol.-Hear. Circ. Physiol.* 2019;317:H330–H344. 10.1152/ajpheart.00054.2019 31125253 PMC6732481

[55] Asli NS : PDGFRα signaling in cardiac fibroblasts modulates quiescence, metabolism and self-renewal, and promotes anatomical and functional repair. *bioRxiv.* 2019;225979. 10.1101/225979

[56] Gao Z : Deletion of the PDGFR-β Gene Affects Key Fibroblast Functions Important for Wound Healing. *J. Biol. Chem.* 2005;280:9375–9389. 10.1074/jbc.M413081200 15590688

[57] Heldin C-H : Targeting the PDGF signaling pathway in tumor treatment. *Cell Commun. Signal.* 2013;11:97. 10.1186/1478-811X-11-97 24359404 PMC3878225

[58] Masamune A Satoh M Kikuta K : Activation of JAK-STAT pathway is required for platelet-derived growth factor-induced proliferation of pancreatic stellate cells. *World J. Gastroenterol.* 2005;11:3385–3391. 10.3748/wjg.v11.i22.3385 15948243 PMC4315992

[59] Heldin C-H Östman A Rönnstrand L : Signal transduction via platelet-derived growth factor receptors. *Biochim. Biophys. Acta (BBA) - Rev. Cancer.* 1998;1378:F79–F113. 10.1016/S0304-419X(98)00015-8 9739761

[60] Yarden Y : Structure of the receptor for platelet-derived growth factor helps define a family of closely related growth factor receptors. *Nature.* 1986;323:226–232. 10.1038/323226a0 3020426

[61] Kohler N Lipton A : Platelets as a source of fibroblast growth-promoting activity. *Exp. Cell Res.* 1974;87:297–301. 10.1016/0014-4827(74)90484-4 4370268

[62] Shook BA : Myofibroblast proliferation and heterogeneity are supported by macrophages during skin repair. *Science.* 2018;362(6417): eaar2971. 10.1126/science.aar2971 30467144 PMC6684198

[63] Wu J-J : The ASIC3-M-CSF-M2 macrophage-positive feedback loop modulates fibroblast-to-myofibroblast differentiation in skin fibrosis pathogenesis. *Cell Death Dis.* 2022;13:527. 10.1038/s41419-022-04981-9 35661105 PMC9167818

[64] Grinnell F Zhu M Carlson MA : Release of Mechanical Tension Triggers Apoptosis of Human Fibroblasts in a Model of Regressing Granulation Tissue. *Exp. Cell Res.* 1999;248:608–619. 10.1006/excr.1999.4440 10222153

[65] Aarabi S : Mechanical load initiates hypertrophic scar formation through decreased cellular apoptosis. *FASEB J.* 2007;21:3250–3261. 10.1096/fj.07-8218com 17504973

[66] Wynn TA : Integrating mechanisms of pulmonary fibrosis. *J. Exp. Med.* 2011;208:1339–1350. 10.1084/jem.20110551 21727191 PMC3136685

[67] Sheng J : M2 macrophage-mediated interleukin-4 signalling induces myofibroblast phenotype during the progression of benign prostatic hyperplasia. *Cell Death Dis.* 2018;9:755. 10.1038/s41419-018-0744-1 29988032 PMC6037751

[68] Hashimoto S Gon Y Takeshita I : IL-4 and IL-13 induce myofibroblastic phenotype of human lung fibroblasts through c-Jun NH2-terminal kinase–dependent pathway. *J. Allergy Clin. Immunol.* 2001;107:1001–1008. 10.1067/mai.2001.114702 11398077

[69] Voulgari PV : Role of Cytokines in the Pathogenesis of Anemia of Chronic Disease in Rheumatoid Arthritis. *Clin. Immunol.* 1999;92:153–160. 10.1006/clim.1999.4736 10444359

[70] Croft M : TNF superfamily in inflammatory disease: translating basic insights. *Trends Immunol.* 2012;33:144–152. 22169337 10.1016/j.it.2011.10.004 PMC3299395

[71] Steele H : TNF superfamily control of tissue remodeling and fibrosis. *Front. Immunol.* 2023;14:1219907. 10.3389/fimmu.2023.1219907 37465675 PMC10351606

[72] Amara N : NOX4/NADPH oxidase expression is increased in pulmonary fibroblasts from patients with idiopathic pulmonary fibrosis and mediates TGFβ1-induced fibroblast differentiation into myofibroblasts. *Thorax.* 2010;65:733–738. 10.1136/thx.2009.113456 20685750 PMC3004009

[73] Serini G : The Fibronectin Domain ED-A Is Crucial for Myofibroblastic Phenotype Induction by Transforming Growth Factor-β1. *J. Cell Biol.* 1998;142:873–881. 10.1083/jcb.142.3.873 9700173 PMC2148176

[74] Meyer-Ter-Vehn T Katzenberger B Han H : Lovastatin inhibits TGF-beta-induced myofibroblast transdifferentiation in human tenon fibroblasts. *Investig. Ophthalmol. Vis. Sci.* 2008;49:3955–3960. 10.1167/iovs.07-1610 18421080

[75] Nakamura R Bing R Gartling GJ : Macrophages alter inflammatory and fibrotic gene expression in human vocal fold fibroblasts. *Exp. Cell Res.* 2022;419:113301. 10.1016/j.yexcr.2022.113301 35931141

[76] Janulaityte I : Asthmatic Eosinophils Alter the Gene Expression of Extracellular Matrix Proteins in Airway Smooth Muscle Cells and Pulmonary Fibroblasts. *Int. J. Mol. Sci.* 2022;23:4086. 10.3390/ijms23084086 35456903 PMC9031271

[77] Kuwabara Y : Role of Matrix Metalloproteinase-2 in Eosinophil-Mediated Airway Remodeling. *Front. Immunol.* 2018;9:2163. 10.3389/fimmu.2018.02163 30294331 PMC6158585

[78] Gallini R Lindblom P Bondjers C : PDGF-A and PDGF-B induces cardiac fibrosis in transgenic mice. *Exp. Cell Res.* 2016;349:282–290. 10.1016/j.yexcr.2016.10.022 27816607

[79] Smith CL Baek ST Sung CY : Epicardial-Derived Cell Epithelial-to-Mesenchymal Transition and Fate Specification Require PDGF Receptor Signaling. *Circ. Res.* 2011;108:e15–e26. 21512159 10.1161/CIRCRESAHA.110.235531 PMC3134964

