# Supplementary material for: Essential growth factor receptors for fibroblast homeostasis and activation: Fibroblast Growth Factor Receptor (FGFR), Platelet Derived Growth Factor Receptor (PDGFR), and Transforming Growth Factor β Receptor (TGFβR)
Source: F1000Res. 2024 May 21;13:120. Originally published 2024 Feb 19. [Version 2] doi: 10.12688/f1000research.143514.2 (PMC11234085; doi:10.12688/f1000research.143514.2)
Supplement: Peer review history

# Reviewer report: Reviewer response for version 2

**DOI:** 10.5256/f1000research.166451.r280957

**Published:** 2024-07-09

**Authors:** Michelle D Tallquist

**Affiliations:** 1 John A. Burns School of Medicine, University of Hawaii at Manoa, Honolulu, USA

The authors have addressed my main concerns.

Is the review written in accessible language?

Yes

Are all factual statements correct and adequately supported by citations?

Partly

Are the conclusions drawn appropriate in the context of the current research literature?

Yes

Is the topic of the review discussed comprehensively in the context of the current literature?

No

Reviewer Expertise:

Fibroblast development, PDGF receptor signaling, fibrosis.

I confirm that I have read this submission and believe that I have an appropriate level of expertise to confirm that it is of an acceptable scientific standard.

# Reviewer report: Reviewer response for version 2

**DOI:** 10.5256/f1000research.166451.r280956

**Published:** 2024-06-12

**Authors:** Natalia Pikor

**Affiliations:** 1 Institute of Immunobiology, Kantonsspital Sankt Gallen (Ringgold ID: 30883), St. Gallen, St. Gallen, Switzerland; 2 ETH Zurich, Zürich, Zurich, Switzerland

The revised version of this manuscript is improved and approved for indexing.

Is the review written in accessible language?

Yes

Are all factual statements correct and adequately supported by citations?

Partly

Are the conclusions drawn appropriate in the context of the current research literature?

Yes

Is the topic of the review discussed comprehensively in the context of the current literature?

Yes

Reviewer Expertise:

Fibroblast immunobiology

I confirm that I have read this submission and believe that I have an appropriate level of expertise to confirm that it is of an acceptable scientific standard.

# Reviewer report: Reviewer response for version 1

**DOI:** 10.5256/f1000research.157187.r252088

**Published:** 2024-03-23

**Authors:** Michelle D Tallquist

**Affiliations:** 1 John A. Burns School of Medicine, University of Hawaii at Manoa, Honolulu, USA

The review by Cheng et al poses the question whether all fibroblast subsets utilize the same growth factors signaling pathways for survival and proliferation. It then discusses three receptor families known to signal in fibroblasts, TGFbR, FGFR, and PDGFR. The review highlights some manuscripts describing the actions of these receptors on a wide range of fibroblast/fibroadipocyte/mesenchymal stem cell populations.

 Comments

 While the title suggests a comprehensive review on this topic, the review focuses on three growth factor receptor families, TGF-bR, FGFR, and PDGFR. Because of the limited focus of the review, I would recommend revision of the title which more closely reflects the scope of the review.

 The review places an emphasis on a dermatopontin expressing fibroblast population (Buechler MB, et al 2021 [Ref 1]).  

 Multiple references focus on mesenchymal stem cells, adipocytes and preadipocytes.  The range of fibroblasts discussed is also very broad. It might be helpful to the reader to understand the connection and definition of these different populations of cells.

 The organ range of fibroblasts discussed is also broad with a mention of prostate, dermis, skeletal muscle, cardiac, pulmonary, kidney, pancreas, and cancer associated fibroblasts.

 Figure 2 is an oversimplification of the receptor pathways illustrated.

 The history of the growth factor receptor’s discovery seems a bit out of the scope of the review.

 Minor comments

 Reference 50 is not Shook et al.

 Reference 49 does not describe lack of PDGFRbeta

Is the review written in accessible language?

Yes

Are all factual statements correct and adequately supported by citations?

Partly

Are the conclusions drawn appropriate in the context of the current research literature?

Yes

Is the topic of the review discussed comprehensively in the context of the current literature?

No

Reviewer Expertise:

Fibroblast development, PDGF receptor signaling, fibrosis.

I confirm that I have read this submission and believe that I have an appropriate level of expertise to confirm that it is of an acceptable scientific standard, however I have significant reservations, as outlined above.

## References

[1] : Cross-tissue organization of the fibroblast lineage. *Nature* .2021;593(7860) : 10.1038/s41586-021-03549-5 575-579 10.1038/s41586-021-03549-5 33981032

# Response

**Published:** 2024-05-12

**Authors:** Maye Cheng

**Affiliations:** Immunology, University of Toronto, Toronto, Ontario, Canada

**We thank both reviewers for dedicating time to provide in-depth and constructive comments for this submission. Their feedback and insight have significantly improved the quality of this review.**

*Reviewer's comments*

 The review by Cheng, et al. poses the question whether all fibroblast subsets utilize the same growth factors signaling pathways for survival and proliferation. It then discusses three receptor families known to signal in fibroblasts, TGFbR, FGFR, and PDGFR. The review highlights some manuscripts describing the actions of these receptors on a wide range of fibroblast/fibroadipocyte/mesenchymal stem cell populations.

 1. While the title suggests a comprehensive review on this topic, the review focuses on three growth factor receptor families, TGF-bR, FGFR, and PDGFR. Because of the limited focus of the review, I would recommend revision of the title which more closely reflects the scope of the review.

**Author Response:**

**We agree with the reviewer’s comment. As suggested, we have revised the title to “** **Essential growth factor receptors for fibroblast homeostasis and activation** **: Fibroblast Growth Factor Receptor (FGFR), Platelet Derived Growth Factor Receptor (PDGFR), and Transforming Growth Factor β Receptor (TGFβR)” to reflect the emphasis of the review.**

 2. The review places an emphasis on a dermatopontin expressing fibroblast population ( Buechler MB, et al 2021 [Ref 1]).  Multiple references focus on mesenchymal stem cells, adipocytes and preadipocytes.  The range of fibroblasts discussed is also very broad. It might be helpful to the reader to understand the connection and definition of these different populations of cells.

**Author Response:**

**We thank the reviewer for their insightful comment. As suggested, we have added definitions of these cells and a discussion of the crosstalk between these populations in lines 113-124.**

 4. The organ range of fibroblasts discussed is also broad with a mention of prostate, dermis, skeletal muscle, cardiac, pulmonary, kidney, pancreas, and cancer associated fibroblasts.

**Author Response:**

**We acknowledge the reviewer’s observation. We included studies across a variety of tissues to broadly examine the effects of growth factor receptors in the body.**

 5. Figure 2 is an oversimplification of the receptor pathways illustrated.

**Author Response:**

**We thank the reviewer for their comment. The reviewer is correct and we have revised the figure to include several additional adaptor molecules and significant pathways.**

 6. The history of the growth factor receptor’s discovery seems a bit out of the scope of the review.

**Author Response:**

**We thank the reviewer for pointing this out. We have removed some portions of the review discussing the history of each receptor.**

Minor comments

 Reference 50 is not Shook et al.

**Author Response:**

**We thank the reviewer for highlighting this. We have inserted the correct reference (62).**

 Reference 49 does not describe lack of PDGFRbeta

**Author Response:**

**We thank the reviewer for highlighting this. We have inserted the correct reference (56).**

# Reviewer report: Reviewer response for version 1

**DOI:** 10.5256/f1000research.157187.r252079

**Published:** 2024-03-20

**Authors:** Natalia Pikor

**Affiliations:** 1 Institute of Immunobiology, Kantonsspital Sankt Gallen (Ringgold ID: 30883), St. Gallen, St. Gallen, Switzerland; 2 ETH Zurich, Zürich, Zurich, Switzerland

In this article, Buechler and colleagues review the literature concerning the influence of key growth factors and cytokines on fibroblast sustenance and activation. In light of the author's seminal contribution to defining Dpt+ universal fibroblasts, an emphasis is placed on the potential relevance of each factor on progenitor fibroblasts.

 The authors have chosen to focus on fibroblast growth factors, platelet-derived growth factors and TGF-beta, in their role for fibroblast homeostasis, maintenance/proliferation and myofibroblastic activation/differentiation. In the introduction an emphasis is placed on universal fibroblasts being a potential progenitor population that can on one hand maintain stemless, and on the other, give rise to both inflammatory fibroblasts and contractile myofibroblasts. Notably, a discussion of the activation factors mediating the differentiation of inflammatory fibroblasts is strikingly missing, despite the wealth of knowledge of tumor necrosis factor superfamily members capable of giving rise to the prototypical inflammatory fibroblast - the fibroblastic reticular cell. Of note, recent studies highlight the presence of the universal progenitor fibroblast population within human and murine lymphoid organs [De Martin A, et.al. 2023 (ref 1), Lütge M, et.al. 2023 (Ref 2)], and several lineage-tracing studies have elucidated the perivascular origin of lymphoid organ FRCs [Cheng HW, et. al. 2019 (ref 3), Prados A, et. al. 2021 (Ref 4)], which is compatible with the paradigm of universal fibroblasts. This section would be a welcome addition to this otherwise comprehensive overview of cues governing fibroblast sustenance and activation.

 As a minor comment on the referenced literature - in its current form the review references 26 other reviews (42% of the references). To better guide a keen reader to the relevant literature, it may be favourable to replace some of the reviews with the main primary papers.

Is the review written in accessible language?

Yes

Are all factual statements correct and adequately supported by citations?

Partly

Are the conclusions drawn appropriate in the context of the current research literature?

Yes

Is the topic of the review discussed comprehensively in the context of the current literature?

Yes

Reviewer Expertise:

Fibroblast immunobiology

I confirm that I have read this submission and believe that I have an appropriate level of expertise to confirm that it is of an acceptable scientific standard, however I have significant reservations, as outlined above.

## References

[1] : PI16+ reticular cells in human palatine tonsils govern T cell activity in distinct subepithelial niches. *Nat Immunol* .2023;24(7) : 10.1038/s41590-023-01502-4 1138-1148 10.1038/s41590-023-01502-4 37202490 PMC10307632

[2] : Conserved stromal-immune cell circuits secure B cell homeostasis and function. *Nat Immunol* .2023;24(7) : 10.1038/s41590-023-01503-3 1149-1160 10.1038/s41590-023-01503-3 37202489 PMC10307622

[3] : Origin and differentiation trajectories of fibroblastic reticular cells in the splenic white pulp. *Nat Commun* .2019;10(1) : 10.1038/s41467-019-09728-3 1739 10.1038/s41467-019-09728-3 30988302 PMC6465367

[4] : Fibroblastic reticular cell lineage convergence in Peyer's patches governs intestinal immunity. *Nat Immunol* .2021;22(4) : 10.1038/s41590-021-00894-5 510-519 10.1038/s41590-021-00894-5 33707780 PMC7610542

# Response

**Published:** 2024-05-12

**Authors:** Maye Cheng

**Affiliations:** Immunology, University of Toronto, Toronto, Ontario, Canada

**We thank both reviewers for dedicating time to provide in-depth and constructive comments for this submission. Their feedback and insight have significantly improved the quality of this review.**

*Reviewer's comments*

 In this article, Buechler and colleagues review the literature concerning the influence of key growth factors and cytokines on fibroblast sustenance and activation. In light of the author's seminal contribution to defining Dpt+ universal fibroblasts, an emphasis is placed on the potential relevance of each factor on progenitor fibroblasts. The authors have chosen to focus on fibroblast growth factors, platelet-derived growth factors and TGF-beta, in their role for fibroblast homeostasis, maintenance/proliferation and myofibroblastic activation/differentiation. In the introduction an emphasis is placed on universal fibroblasts being a potential progenitor population that can on one hand maintain stemless, and on the other, give rise to both inflammatory fibroblasts and contractile myofibroblasts.

 1. Notably, a discussion of the activation factors mediating the differentiation of inflammatory fibroblasts is strikingly missing, despite the wealth of knowledge of tumor necrosis factor superfamily members capable of giving rise to the prototypical inflammatory fibroblast - the fibroblastic reticular cell.

**We thank the reviewer for mentioning this important comment. We have** **referenced the importance of TNFSF members in promoting activation of inflammatory fibroblasts in lines 489-492. We feel that the contribution of TNFSF members is out of the scope of this review as we focused here on FGFR, PDGFR, and TGFβR. Additionally, TNFSF members and their association with fibroblasts have been recently reviewed in Steele *et al. *(2023).**

 2. Of note, recent studies highlight the presence of the universal progenitor fibroblast population within human and murine lymphoid organs [De Martin A, et.al. 2023 (ref 1), Lütge M, et.al. 2023 (Ref 2)], and several lineage-tracing studies have elucidated the perivascular origin of lymphoid organ FRCs [Cheng HW, et. al. 2019 (ref 3), Prados A, et. al. 2021 (Ref 4)], which is compatible with the paradigm of universal fibroblasts. This section would be a welcome addition to this otherwise comprehensive overview of cues governing fibroblast sustenance and activation.

**We appreciate the reviewer’s comments. We have included remarks** **(lines 125-130)** **on the recent studies examining *Pi16+ *fibroblasts in SLOs as an additional rationale for examining the growth factor requirements for *Dpt+ *fibroblasts. We have also reviewed and included a section on the origin of FRCs in SLOs in lines 105-112.**

 3. As a minor comment on the referenced literature - in its current form the review references 26 other reviews (42% of the references). To better guide a keen reader to the relevant literature, it may be favourable to replace some of the reviews with the main primary papers.

**We agree with the reviewer’s insight. We have added additional references from primary sources. Currently, this submission contains 32% of references from other reviews.**
